# Matrix metalloproteinase-9 involvement in the structural plasticity of dendritic spines

**DOI:** 10.3389/fnana.2014.00068

**Published:** 2014-07-10

**Authors:** Michal Stawarski, Marzena Stefaniuk, Jakub Wlodarczyk

**Affiliations:** ^1^Laboratory of Cell Biophysics, Department of Molecular and Cellular Neurobiology, Nencki Institute of Experimental BiologyWarsaw, Mazowieckie, Poland; ^2^Laboratory of Neurobiology, Department of Molecular and Cellular Neurobiology, Nencki Institute of Experimental BiologyWarsaw, Mzowieckie, Poland

**Keywords:** matrix metalloproteinase-9, dendritic spines, structural synaptic plasticity, extracellular matrix, epilepsy

## Abstract

Dendritic spines are the locus for excitatory synaptic transmission in the brain and thus play a major role in neuronal plasticity. The ability to alter synaptic connections includes volumetric changes in dendritic spines that are driven by scaffolds created by the extracellular matrix (ECM). Here, we review the effects of the proteolytic activity of ECM proteases in physiological and pathological structural plasticity. We use matrix metalloproteinase-9 (MMP-9) as an example of an ECM modifier that has recently emerged as a key molecule in regulating the morphology and dysmorphology of dendritic spines that underlie synaptic plasticity and neurological disorders, respectively. We summarize the influence of MMP-9 on the dynamic remodeling of the ECM via the cleavage of extracellular substrates. We discuss its role in the formation, modification, and maintenance of dendritic spines in learning and memory. Finally, we review research that implicates MMP-9 in aberrant synaptic plasticity and spine dysmorphology in neurological disorders, with a focus on morphological abnormalities of dendritic protrusions that are associated with epilepsy.

## Introduction

Structural plasticity is an active field in neuroscience, with pivotal implications for the understanding of many different levels of learning and memory and a wide range of neurological and cognitive disorders (Sala and Segal, 2014; Penzes et al., 2011). Dendritic spines are plastic structures that undergo morphological changes in response to stimuli that modulate neuronal activity. Such remodeling underlies the formation and long-term storage of information in the brain, and spine remodeling frequently accompanies neurodegenerative diseases, i.e., ischemia (Brown et al., 2008b) or spinal cord injury (Kim et al., 2006) lead to a reduction in spine density and elongation of the remaining spines; traumatic brain injury alters dendritic spines stability (Campbell et al., 2012). The extracellular matrix (ECM) of the brain mediates structural stability by creating a scaffold for dendritic spines (de Vivo et al., 2013). ECM components do not constitute only a passive environment. Some ECM components (e.g., matrix metalloproteinase-9 [MMP-9]) also actively participate in synaptic plasticity. Growing evidence indicates a particular role for MMP-9 in the mediation of structural plasticity in the brain.

In the present review, we discuss the concept of the tetrapartite synapse, with a particular emphasis on ECM proteins, and highlight both the beneficial and detrimental roles of MMP-9 in pathological structural brain plasticity. We focus on epileptogenesis as an example of a disease in which the role of MMP-9 in aberrant synaptic plasticity and permanent impairment is particularly significant (Wilczynski et al., 2008).

## Tetrapartite synapse

The first high-resolution observations of neurons by Ramón y Cajal unveiled the existence of dendritic spines, small membranous protrusions on the surface of dendrites (Ramón y Cajal, 1888, 1899). Ramón y Cajal theorized that they could be involved in signal transmission within the brain (Ramón y Cajal, 1891, 1893), giving rise to the classical theory of a dipartite synapse as a basic unit of neuronal information processing. The dipartite synapse is formed by presynaptic and postsynaptic parts that are found on presynaptic boutons and dendritic spines, respectively, separated by the synaptic cleft. In the decades that followed, research showed that this model was overly simplified, and additional players were added to the picture. The concept of a tripartite synapse appeared, in which the astrocyte, once believed to be an inert, “neuron-feeding” cell, actively participates in synaptic transmission (Araque et al., 1999). Soon new components of the synapse were discovered, giving rise to the tetrapartite or even pentapartite concept of the synapse. In one conceptualization, microglia (reviewed in De Leo et al., 2006) join pre- and postsynaptic neurons and astrocytes to form the synapse. In another conceptualization, the ECM is added as a key component of the synapse (reviewed in Dityatev and Rusakov, 2011). The ECM is a complex protein network that fills the extracellular space and is secreted by neurons, glia, and non-neuronal cells. It was previously seen as an inert component of the synapse, a scaffold that maintains synaptic integrity, with no effect on synaptic transmission. The reality, however, turned out to be quite different. Recent research has shown that the ECM actively regulates a plethora of cellular functions, from the initial establishment of the synapse to the regulation of synaptic transmission and synaptic plasticity (reviewed in Dityatev et al., 2010). The ECM has begun to be recognized as a critical factor that affects synapses by enveloping them and forming a synaptic element. ECM components with known roles in the regulation of synaptic transmission include laminin, tenascins, thrombospondin, lectins, and MMPs, to name a few (comprehensively reviewed in Dansie and Ethell, 2011). Among the numerous ECM components that have been proposed to play roles in brain plasticity, MMP-9 has recently emerged as a key molecule involved in long-term memory and the underlying synaptic changes (Rivera et al., 2010; Huntley, 2012; Tsien, 2013). The focal point of synaptic changes within the brain are dendritic spines that harbor synaptic contacts. Their stability is correlated with their shape, in which mushroom-like spines are generally more stable than thin, long spines. The size of the spine head is well known to be correlated with the area of the postsynaptic density (PSD; Harris and Stevens, 1989; Meyer et al., 2014) and AMPA receptor number (Nusser et al., 1998; Kharazia and Weinberg, 1999; Takumi et al., 1999; Szepesi et al., 2014). Dendritic spine neck length on the other hand is correlated with postsynaptic potential (Araya et al., 2006; Tønnesen et al., 2014). Thus, dendritic spine morphology has been accepted to determine the strength of synaptic connections. The structural plasticity of dendritic spines is widely seen as the basis of the primary functions of the central nervous system, including learning and memory. The influence of MMP-9 on dendritic spine morphology makes it a perfect candidate molecule for synaptic remodeling.

## Matrix metalloproteinase-9

MMP-9 is a 92 kDa protein that belongs to a family of zinc- and calcium-dependent endopeptidases. Because of its ability to cleave gelatin, it is classified as a gelatinase. It is encoded in the human genome by a gene located on chromosome 20 (20q13.12). The molecular biology of MMP-9 was summarized in an excellent, exhaustive review by Vandooren et al. (2013).

MMP-9 has a complex domain structure, with a signal peptide at the N-terminus, followed by a propeptide, a catalytic domain with a zinc ion binding site, three fibronectin type II inserts, a proline-rich and heavily *O*-glycosylated linker, and a hemopexin domain located at the C-terminus of the protein (Stute et al., 2003). The propeptide contains an evolutionarily conserved PRCGVPDV domain that binds the zinc ion in the catalytic domain and blocks the activity of the enzyme until it is cleaved (Van Wart and Birkedal-Hansen, 1990; Becker et al., 1995). The overlapping substrate specificity of MMPs (to date, 25 MMPs have been identified in humans) is attributable to a zinc-binding motif, HExGHxxGxxH (where x signifies any amino acid), within the catalytic domain, which is shared by all MMPs. Tandemly repeated fibronectin type II inserts within the catalytic domain are responsible for gelatin binding. The linker allows for independent movement of the catalytic and hemopexin domains, which influence enzyme conformation (Rosenblum et al., 2007) and the substrate specificity of MMP-9. The hemopexin domain is able to bind an endogenous MMP-9 inhibitor called tissue inhibitor of matrix metalloproteinases-1 (TIMP-1) and several MMP-9 substrates. It is also responsible for the membrane docking of MMP-9 (Bode et al., 1999) and formation of homo- and heterodimers with neutrophil gelatinase-associated lipocalin (NGAL; Kjeldsen et al., 1993; Cha et al., 2002).

MMP-9 is ubiquitously expressed throughout the body. Within the resting brain, it is mostly synthesized by neurons but to some extent also by glia in such structures as the hippocampus, cerebral cortex, and cerebellum. It is extracellularly secreted, although recent studies have also revealed its presence in the nucleus of muscle cells (Yeghiazaryan et al., 2012), neurons (Yang et al., 2010; Hill et al., 2012), human glial cells (Pirici et al., 2012), and mitochondria of retinal capillary cells. MMP-9 may act as a negative regulator of mitochondrial function and may be involved in apoptosis (Kowluru et al., 2011).

The expression of MMP-9 is regulated on multiple levels: (1) transcription (for a comprehensive review of the transcriptional and epigenetic regulation of MMP-9 expression, including regulation through non-coding RNAs, see Labrie and St-Pierre, 2013; several studies also indicate that reactive oxygen species (ROS) activate MMP-9 expression through AP-1 transcription factors; Hasebe et al., 2007; Hsieh et al., 2014); (2) posttranslation (also involving non-proteolytic activation); (3) local translation (Dziembowska et al., 2012); (4) sequestration on the cell membrane (e.g., binding to cell adhesion molecules, such as hyaluronian receptor CD44 (Bourguignon et al., 1998), integrins (Wang et al., 2003), lipoprotein receptor-related protein-1 (LRP-1), and megalin/LRP-2 (Van den Steen et al., 2006)); (5) internalization (Hahn-Dantona et al., 2001); and (6) delayed activation that involves cleavage of the propeptide and co-secretion with TIMP-1, its endogenous inhibitor (Sbai et al., 2010). In the brain, the primary transcriptional regulators of MMP-9 expression are AP-1 and nuclear factor-κB (NF-κB). Yin Yang 1 (YY1) was identified as a strong repressor of MMP-9 transcription in the rat hippocampus *in vivo* and cultured neurons (Rylski et al., 2008).

MMP-9 activation usually occurs through the cleavage of a propeptide that disrupts its zinc ion binding properties. Cleavage may also be performed by other MMPs and the tissue plasminogen activator (tPA)-plasmin system (Bruno and Cuello, 2006). However, non-proteolytic activation/inactivation is also possible through posttranslational modification. The thiol modification of methionine and cysteine residues within the catalytic domain and nitration or oxidation of the propeptide cysteine that is responsible for zinc ion binding are also able to activate MMPs without propeptide removal. Finally, nitric oxide, a commonly occurring secondary messenger in the brain, is able to regulate the stability of MMP-9 mRNA (Akool et al., 2003).

MMP-9 and TIMP-1 are secreted by neurons in a Golgi-dependent manner in 160–200 nm vesicles. The vesicles move along microtubules and microfilaments. They are distributed in the somatodendritic compartment and can be found in dendritic spines (Sbai et al., 2008).

MMP-9 regulates numerous cell activities through its involvement in matrix remodeling and the liberation of macromolecules (e.g., growth factors) that are embedded within the ECM. MMP-9 is involved in various physiological functions, such as tissue remodeling, cellular differentiation (Zimowska et al., 2013), cell-cell contact and cell migration (Kim et al., 2012), the release of cytokines and regulation of growth factor activity (Schonbeck et al., 1998), survival and apoptosis (Kowluru et al., 2011), angiogenesis, inflammation, and signaling (for a comprehensive review of MMP-9 function, see Vandooren et al., 2013; Verslegers et al., 2013). Aside from maintaining tissue homeostasis, MMP-9 plays a role in a range of pathologies (comprehensively reviewed in Rivera et al., 2010; Kaczmarek, 2013).

Polymorphisms that have been identified in the MMP-9 gene promoter ([CA]n microsatellite at position -90 and SNP at -1562) that affect the binding of nuclear proteins and thus the expression levels of the protein were correlated with an increased incidence of several pathologies in human populations (Ye, 2000). Additionally, a functional polymorphism that might affect the binding of non-coding RNAs and thus mRNA transport and translation was identified within the 3’ untranslated region of MMP-9 mRNA (Yuan et al., 2013).

**Figure 1:**
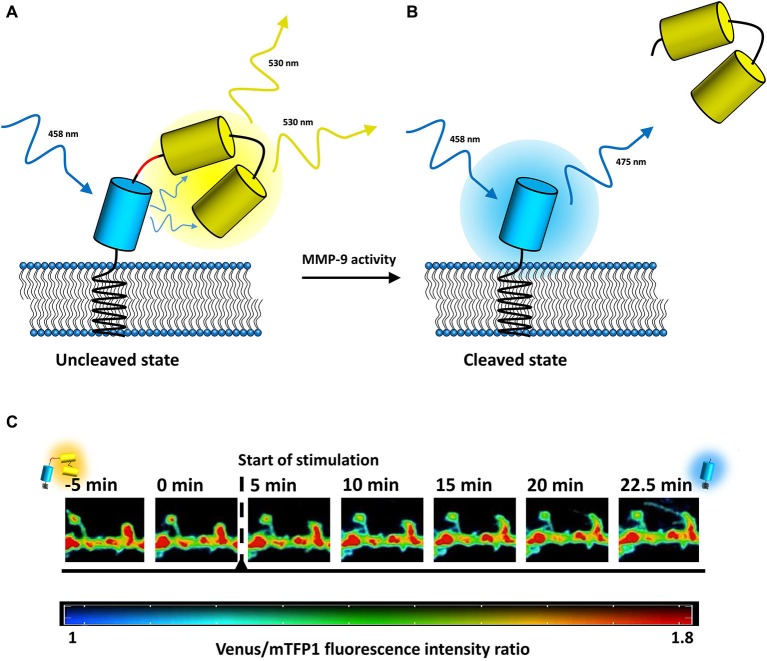
**MMP-9 activity detection mechanism of the biosensor.** **(A)** In an uncleaved state, the excitation energy is transferred from mTFP1 (depicted in blue) to Venus (depicted in yellow), resulting in the fluorescence of the yellow fluorescent protein. **(B)** Once MMP-9 cleaves the biosensor (sequence recognized by MMP-9 is marked in red), the Venus proteins are released, the FRET phenomenon disappears, and the fluorescence of mTFP1 increases. **(C)** Time-lapse imaging of endogenous MMP-9 activity visualized with the MMP-9 activity biosensor expressed in a primary rat hippocampal culture. Colors indicate the value of Venus-to-mTFP1 fluorescence intensity ratio (reds—high values of the ratio/uncleaved biosensor; blues—low values of the ratio/cleaved biosensor; see the color bar below image insets). Stimulation was performed with the chemical long-term potentiation (LTP) protocol (see Stawarski et al., 2014). Biosensor pictures in the left and right of the diagram indicate visually the state of the biosensor (uncleaved/cleaved).

The monitoring of MMP-9 activity originally took advantage of its gelatinolytic properties in the form of zymography, in which MMP-9 cleaves an FITC-tagged gelatin (DQ-gelatin). Under normal conditions, FITC fluorescence is almost completely quenched unless DQ-gelatin is cleaved by MMP-9. In such a case, fluorescence increases and can be readily monitored using confocal microscopy. However, DQ-gelatin does not enable the tracking of MMP-9 activity with high spatial and temporal resolution because it is freely diffusive. Moreover, DQ-gelatin is also cleaved by MMP-2, which generally has a much higher level of expression than MMP-9. Therefore, assays that utilize DQ-gelatin, with the exception of gel zymography, are highly nonspecific. In recent years, a number of new MMP-9 activity biosensors have been developed (Faust et al., 2008; Fudala et al., 2011; Akers et al., 2012; Gustafson et al., 2013) in response to the perceived shortcomings of classical zymographic approaches. The potential of MMP-9 as a prognostic marker of cancer led to considerable interest in developing diagnostic and analytical probes to detect the proteolytic activity of MMP-9 in cancer (for a survey of several MMP-9 activity probes used in cancer detection, see Roy et al., 2011; for a review of MMP-9 detection methods in cancer, see Scherer et al., 2008; for MMP-9 near-infrared fluorescence probes in *in vivo* imaging, see Wallis de Vries et al., 2009; Kaijzel et al., 2010; Akers et al., 2012; Lee et al., 2012). We recently developed a genetically encoded fluorescence resonance energy transfer (FRET)-based MMP-9 activity biosensor (Stawarski et al., 2014) that is compatible with live cell imaging approaches and can be used to study the effects of MMP-9 on structural plasticity with very high spatiotemporal resolution. It is membrane-anchored and utilizes the teal fluorescent protein (mTFP1) as a donor of energy and two tandemly repeated Venus proteins as energy donors to increase the resonant energy transfer level (Figure 1). The biosensor was engineered for the highest possible FRET efficiency and incorporates a synthetic MMP-9 cleavage site within an α-helical region, giving the biosensor high sensitivity to MMP-9 action and improved specificity. The biosensor can be used to study the action of MMP-9 at the level of single dendritic spines, providing an opportunity to unambiguously correlate endogenous MMP-9 activity with the plastic changes of dendritic spines. Furthermore, by combining the biosensor with one of several recently described brain optical clearing techniques (several optical clearing agents were recently reviewed in Zhu et al., 2013; see also Chung et al. (2013) for CLARITY, Hama et al. (2011) for Scale and Susaki et al. (2014) for CUBIC) bridging the gap between studies of the global anatomical changes that occur because of synaptic plasticity and locally regulated extremely low-level proteolytic activity around single neurons might be possible, thus providing insights into the basic mechanisms of brain plasticity.

## MMP-9 in physiological structural plasticity

Research on MMP-9 was originally concentrated on its role in the pathology of the central nervous system (e.g., post-injury and post-stroke damage to brain tissue) due to its ECM-cleavage properties. However, the first indication that MMP-9 may play a role beyond the pathology of the brain came from research on the kainate-induced epilepsy models in mice. Kainate-induced seizures lead to massive cell death in CA regions of the hippocampus, the limbic cortex, and the amygdala but a pronounced plasticity in the dentate gyrus (DG; Zagulska-Szymczak et al., 2001). Nedivi et al. (1993) demonstrated that TIMP-1 mRNA (i.e., an endogenous inhibitor of MMPs, including MMP-9) is upregulated following kainate-induced seizures. A follow-up study (Szklarczyk et al., 2002) revealed that MMP-9 mRNA, protein, and activity levels are also upregulated in the DG of the hippocampus under the same experimental conditions. Furthermore, Jaworski et al. (1999) observed that the expression of TIMP-1 is coupled to neuronal excitation and spatially and temporarily overlaps with c-Fos expression. Kuzniewska et al. (2013) and Ganguly et al. (2013) also demonstrated that MMP-9 expression depends on the c-Fos transcriptional regulation. C-Fos role in brain plasticity was postulated already by Kaczmarek et al. (2002). Nagy et al. (2006) and Bozdagi et al. (2007) demonstrated that MMP-9 is a necessary component of long-term potentiation (LTP; i.e., an experimental paradigm that mimics certain aspects of physiological plasticity) both in acute hippocampal slices and *in vivo* in urethane-anesthetized rats. They discovered that MMP-9 is required in the late phase of LTP in the CA1 field, and MMP-9 inhibition by whatever means (e.g., inhibitors and antisense RNA) leads to a rapid return of synaptic potentiation to baseline levels. Research on MMP-9 knockout mice revealed diminished LTP that could be rescued by exogenously applied recombinant MMP-9 (Nagy et al., 2006). Meighan et al. (2006) demonstrated that spatial learning leads to alterations in MMP-9 mRNA and protein levels. The injection of either the broad-spectrum MMP inhibitor FN-439 or antisense RNA led to markedly diminished learning in the Morris water maze. A similar effect was achieved with the *N*-methyl-D-aspartate (NMDA) receptor antagonist MK801. Finally, Meighan et al. (2006) demonstrated that the effect of MMPs on learning is facilitated through changes of the actin cytoskeleton. Recent studies also revealed that MMP-9 is required for cortical plasticity evoked by sensory deprivation (e.g., whisker plucking (Kaliszewska et al., 2012) and monocular deprivation (Spolidoro et al., 2012)) and LTP in the mossy fiber-CA3 pathway (Wiera et al., 2012, 2013). Physiological remodeling is also strongly influenced by experience-dependent mechanisms that imply an interaction between neural circuits and the external world. An enriched environment has been shown to influence brain plasticity by inducing MMP-9 activity (Foscarin et al., 2011; Cao et al., 2014). More information on the role of MMP-9 in physiological plasticity can be found in recently published reviews (Rivera et al., 2010; Wlodarczyk et al., 2011).

**Figure 2:**
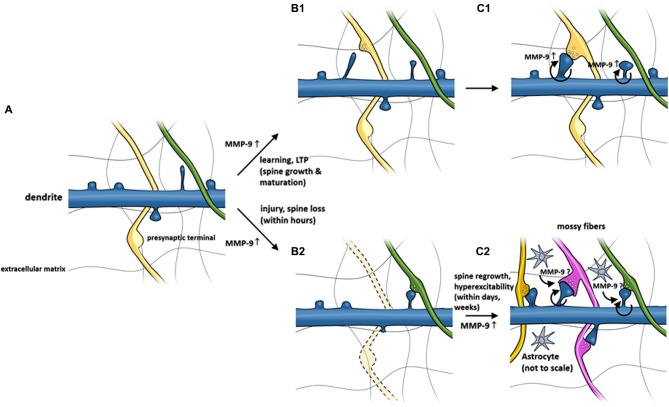
**MMP-9 involvement in morphological changes of dendritic spines in LTP and possible role in epileptogenesis-related spine pathology.** **(A)** Dendritic spines extend as small protrusions from dendritic shaft. **(B1)** MMP-9 level is elevated upon stimulus which leads to spine elongation and **(C1)** maturation. **(B2)** Fast (within minutes) activation of MMP-9 by injury or seizures may induce spine loss. **(C2)** Continuous increase of MMP-9 (secondary to seizures, within days) may cause regrowth of dendritic spines and contribute to long-term circuitry remodeling underlying seizures.

Matrix metalloproteinases are functionally involved in the regulation of synaptic plasticity (Nagy et al., 2006; Bozdagi et al., 2007; Okulski et al., 2007; Rivera et al., 2010; Huntley, 2012; Szepesi et al., 2013) and formation and maintenance of dendritic spines (Wang et al., 2008; Bilousova et al., 2009; Michaluk et al., 2011). Although details of MMP-9 action on dendritic spines are not fully elucidated, the research performed so far allowed to construct a model of MMP-9 activity around dendritic spines, which is presented in Figure 2. The model postulates that MMP-9 is released from dendritic spines following a stimulus. MMP-9 activity, possibly mediated through β1 integrin signaling causes the elongation of dendritic spines and increases the mobility of NMDA receptors. The time window in which MMP-9 is active is brief, as it is almost immediately inactivated by its endogenous inhibitor TIMP-1. MMP-9 inhibition allows the dendritic spines to mature, incorporate AMPA receptors and form active synapses. The model presented above is supported by numerous observations, which are briefly summarized in the next paragraph.

The presence of MMP-9-coding mRNA, protein, and enzymatic activity at the level of dendritic spines was confirmed by recent studies (Konopacki et al., 2007; Wilczynski et al., 2008; Gawlak et al., 2009; Dziembowska et al., 2012). The activity-dependent local translation of MMP-9 mRNA and protein release was recently demonstrated by Dziembowska et al. (2012), supporting the hypothesis that locally secreted MMP-9 and other dendritically synthesized proteins are involved in the structural and functional plasticity of activated synapses. MMP-9 also increases the lateral mobility of NMDARs (Michaluk et al., 2009). Michaluk et al. (2011) also demonstrated that elongation and spine thinning are regulated by integrin β1 signaling and are accompanied by changes in the decay time of miniature synaptic currents. The blockade of integrin β1 signaling with an integrin β1 antibody abolished both the MMP-9-dependent increase in NMDAR mobility and its ability to affect spine morphology. The ICAM-5 ectodomain produced by MMP-9 cleavage stimulates an increase in AMPA mini excitatory postsynaptic current (mEPSC) frequency and leads to recruitment of the AMPAR GluA1 subunit to the membrane (Lonskaya et al., 2013).

The activity of MMP-9 that was locally released in response to stimuli was also recently reported to modulate the morphology of dendritic spines (Wang et al., 2008; Bilousova et al., 2009). Admittedly, there is some confusion on how exactly MMP-9 affects the spine morphology, with different groups reporting markedly different effects, with some groups observing spine maturation following MMP-9 activity, while others report dendritic spine elongation. Wang observed that MMP-9 was required in spine enlargement associated with LTP in acute hippocampal slices. That observation indicates that MMP-9 may drive the establishment of persistent modification of both synapse structure and function (Wang et al., 2008). Intracellular adhesion molecule-5 (ICAM-5) cleavage by MMP-9 causes the elongation of dendritic filopodia in dissociated neuronal cultures (Tian et al., 2007) and influences AMPAR-dependent glutamatergic transmission. Our research indicates that the enzymatic activity of MMP-9 is able to cause an elongation and thinning of dendritic spines on hippocampal neurons in three experimental models: transgenic rats that overexpress auto-activating MMP-9, dissociated hippocampal cultures, and organotypic cultures (Michaluk et al., 2011). Bilousova observed that the incubation of dissociated neuronal cultures with recombinant MMP-9 led to the transformation of mushroom-shaped dendritic spines into filopodia-like protrusions (Bilousova et al., 2009). Minocycline, a drug whose pleiotropic effects include the inhibition of MMP-9 activity, reverses filopodia transformation toward mature spines in an animal model of fragile X syndrome (Fmr1 knockout mice). Apparently conflicting effects of MMP-9 on spine structure and dynamics can be explained by different research protocols, including enzyme concentration, treatment duration, and age and developmental stage of the neurons. The research discussed above also differs in the manner by which MMP-9 was applied (i.e., bath application *vs*. local application), the duration of MMP-9 activity influenced by the inhibitory effect of TIMP-1 (an endogenous MMP-9 inhibitor), and the maturity of neurons. Wang et al. (2008) observed that MMP-9 promotes the maturation of dendritic spines, whereas Tian et al. (2007) and Michaluk et al. (2011) reported something markedly different (i.e., MMP-9 or MMP-9-released products stimulated spine elongation). Wang et al. (2008) performed their studies using acute slices from postnatal day 14 (PD14) to PD21 rats, whereas Michaluk et al. (2011) chose an organotypic culture model from PD7 rats. Furthermore, Wang et al. (2008) applied MMP-9 locally with a stimulating electrode, which more closely resembles physiological conditions, in contrast to the study by Michaluk et al. (2011), who used bath application, which is closer to pathological conditions. MMP-9 activity is thus believed to be strictly regulated, local, and transient, and the inhibition of MMP-9 may be the final step in spine maturation, in which a filopodium-like protrusion matures into a mushroom-like dendritic spine (Tian et al., 2007; Bilousova et al., 2009). Studying the effect of TIMP-1 sequestration on spine morphology might be advantageous.

Spine head protrusions (SHPs) are small filopodia-like processes that extend from the dendritic spine head. They represent a new type of postsynaptic structural remodeling that follows neuronal activity. We recently reported the role of MMP-9 in the formation of SHPs (Szepesi et al., 2013). Chemically induced LTP (cLTP) leads to the upregulation of MMP-9 activity in dissociated hippocampal cultures and appears to control SHP formation. The growth of SHPs is abolished by inhibiting MMP activity or influencing microtubule dynamics. Recombinant auto-activating MMP-9 promotes the formation of SHPs in organotypic hippocampal slices. Spines with SHPs were also shown to recruit new postsynaptic AMPA receptors following cLTP, and AMPAR recruitment was MMP-dependent.

Research indicates that the effect of MMP-9 on spine morphology may be mediated not by a general disruption of ECM structure (Michaluk et al., 2009) but rather by the tightly regulated cleavage of specific ECM proteins that would then trigger the intracellular integrin signaling pathway (Wang et al., 2008; Michaluk et al., 2011). The modifications of spine morphology appear to involve MMP-9 cleavage of the following proteins: β-dystroglycan, ICAM-5, integrins (MMP-9 is known to cleave integrin β2; Vaisar et al. (2009), and β4 (Pal-Ghosh et al., 2011). Indirect evidence also suggests that MMP-9 may drive the cleavage of integrin β1 (Kim et al., 2009), neuroligin-1, and ephrin (Nagy et al., 2006; Michaluk et al., 2007, 2009; Tian et al., 2007; Conant et al., 2010; Peixoto et al., 2012). The MMP-9-driven cleavage of ICAM-5, a negative regulator of spine maturation, was observed following neuronal stimulation and led to spine maturation (Tian et al., 2007; Conant et al., 2010). Peixoto et al. (2012) observed that neuroligin-1 cleavage by MMP-9 at the postsynaptic site of glutamatergic synapses resulted in a destabilized presynaptic site and modification of synaptic transmission. The Ephrin/Eph receptor complex, cleaved by MMP-9 following hippocampal LTP, is involved in learning and memory (Klein, 2004; Murai and Pasquale, 2004). Therefore, MMP-9 is clearly an important player in the dynamic remodeling of dendritic spines, and its affinity for numerous proteins supports the concept of a tetrapartite synapse (Dityatev and Rusakov, 2011).

Matrix metalloproteinases also process various non-ECM proteins, such as growth factor precursors, cell-surface receptors, and adhesion molecules (Nagase and Woessner, 1999; Ethell and Ethell, 2007; Michaluk and Kaczmarek, 2007). Several MMP-9 substrates were identified only *in vitro* (e.g., the ECM and the basement membrane molecules brevican, laminin and aggrecan; Nakamura et al., 2000; Overall, 2002; Morrison et al., 2009) and not confirmed *in vivo*. β-dystroglycan (β-DG) was confirmed to be cleaved by MMP-9 *in vivo* in response to enhanced neuronal activity (Michaluk et al., 2007). β-DG is a transmembrane protein that serves as a cell-surface anchor for α-dystroglycan, which binds to the extracellular domains of β-DG and laminin, agrin, and perlecan (Ervasti and Campbell, 1993; Gee et al., 1993; Bowe et al., 1994; Campanelli et al., 1996; Peng et al., 1998; Henry et al., 2001) in non-neuronal cells and neurexins in the central nervous system (Sugita et al., 2001). Thus, MMP-9 activity may uncouple the cytoskeleton from the ECM. Other cell-surface receptors that were confirmed to be MMP-9 substrates include the interleukin-2 (IL-2) receptor α chain (CD25; Sheu et al., 2001; De Paiva et al., 2009), ephrin B (Lin et al., 2008), and NG2 proteoglycan in the spinal cord (Larsen et al., 2003).

Bajor et al. (2012) isolated two synaptic proteins that are cleaved *in vivo* by MMP-9: synaptic cell adhesion molecule-2 (synCAM-2) and collapsin response mediator protein-2 (CRMP-2). CRMP-2 is involved in axon guidance, neurite outgrowth, and the regulation of neuronal differentiation (Inagaki et al., 2001; Fukata et al., 2002; Yoshimura et al., 2005). It was suggested to play a role in numerous neurological diseases, such as Alzheimer’s disease, epilepsy, and ischemia (Gu and Ihara, 2000; Czech et al., 2004; Uchida and Goshima, 2005). SynCAM-2 (also known as Necl-3, IgSF4D, and Cadm-2) is a Ca^2+^-binding immunoglobulin-like transmembrane protein that is expressed by neurons in the developing and adult brain. It is localized to the synaptic cleft and contributes to synaptic organization and function (Fogel et al., 2007).

Growth factors and signaling molecules that were confirmed to be processed by MMP-9 *in vivo* include IL-8 (Van den Steen et al., 2000), pro brain-derived neurotrophic factor (BDNF; Hwang et al., 2005; Mizoguchi et al., 2011a), pro tumor necrosis factor α (TNF-α; Roghani et al., 1999), and pro transforming growth factor β (TGF-β; Yu and Stamenkovic, 2000). The ability of MMP-9 to cleave β-amyloid peptide is particularly interesting because an increase in MMP-9 expression was observed in Alzheimer’s disease patients by several research groups (Backstrom et al., 1996; Yan et al., 2006; Mizoguchi et al., 2011a; Filippov and Dityatev, 2012).

## MMP-9 in aberrant structural plasticity

Accumulating evidence suggests that MMP-9 may also play a relatively direct role in other forms of learning and memory, including those associated with addiction. Many drugs and alcohol induce neuroplastic changes in pathways that subserve emotion and cognition. Elevated MMP-9 activity has been implicated in the synaptic remodeling that is important for the reactivation of cocaine memory in rats (Brown et al., 2008a). In mice, methamphetamine treatment resulted in the rapid upregulation of MMP-9 (Liu et al., 2008; Conant et al., 2011). MMP-9 has also been implicated in the plastic changes induced by alcohol addiction (Samochowiec et al., 2010). Importantly, both drug and chronic alcohol exposure cause structural alterations of dendrites and their spines (Zhou et al., 2007; Shen et al., 2009).

The importance of MMP-9 in shaping dendritic spine architecture in disease is further supported by autism and mental retardation studies. Fragile X syndrome is a form of inherited intellectual disability (Hagerman et al., 2005; Bagni et al., 2012). Patients with fragile X syndrome have aberrant dendritic spine morphology (Rudelli et al., 1985). Long and thin immature dendritic spines are also observed in Fmr1 knockout mice, a model of fragile X syndrome (Comery et al., 1997). MMP-9 has been shown to be highly increased in the fragile X syndrome mouse model (Bilousova et al., 2009). The reduction of MMP-9 levels induced by minocycline promoted dendritic spine maturation and improved general behavioral performance. High MMP-9 activity levels are also lowered by minocycline in fragile X syndrome patients (Dziembowska et al., 2013). Notably, minocycline has been tested in clinical trials to treat fragile X syndrome and shown to provide significant functional benefits (Paribello et al., 2010; Utari et al., 2010; Leigh et al., 2013). Matrix metalloproteinases have also been implicated in other forms of autism (Abdallah and Miche1, 2013). MMP-9 levels are elevated in patients with Autism Spectrum Disorder (Abdallah et al., 2012). MMP-9 levels are also increased in patients with schizophrenia (Rybakowski et al., 2013), and dendritic spine alterations have been identified in multiple brain regions in schizophrenia (Glausier and Lewis, 2013).

Aberrant structural plasticity is a major phenomenon associated with epilepsy (Scharfman, 2002). Epilepsy is a brain disorder characterized by an enduring predisposition to the generation of epileptic seizures, understood as sudden excessive neuronal discharges. In many cases, epilepsy develops as a result of brain damage caused by traumatic brain injury, stroke, or infection (Banerjee and Hauser, 2008). The primary insult is associated with an increased incidence of secondary injuries that can develop within hours to days or even weeks. Secondary brain injury is thought to be responsible for the development of many of the aforementioned sustained neurological deficits (Bolkvadze and Pitkanen, 2012; Rezaei et al., 2012; Mollayeva et al., 2013). Patients with epilepsy frequently suffer from memory impairment, behavioral problems, and psychiatric disorders (Dodrill, 2002; Helmstaedter, 2002; Williams, 2003; Elger et al., 2004; Berg, 2011). Dendritic spines receive a majority of excitatory synaptic inputs. Transmission and dendritic spine shape and function are strictly related, and such structural changes likely constitute hallmarks of pathology and the observed cognitive deficits.

Indeed, abnormalities in dendritic spines have been commonly associated with human epilepsy and animal models of epilepsy (Swann et al., 2000; Wong, 2005). A prominent decrease in dendritic spine density has been observed in pyramidal neurons in the hippocampus and neocortex and dentate granule cells in patients with temporal lobe epilepsy (Multani et al., 1994; Jiang et al., 1998; Blümcke et al., 1999; Freiman et al., 2011; Kitaura et al., 2011). Similar aberrations have been observed in animal models of epilepsy (Jiang et al., 1998; González-Burgos et al., 2004; Ampuero et al., 2007). Notably, other alterations, such as an increase in dendritic spine number or size, have also been reported (Represa et al., 1993; Suzuki et al., 1997; Isokawa, 2000). These common features of both human and animal studies indicate that dendritic spine abnormalities represent an important factor in the pathological mechanisms of posttraumatic epilepsy, but it is still unclear whether they are more related to the cause or consequence of seizures (Wong and Guo, 2013). Furthermore, dendritic spine pathology may be both the cause and consequence of seizures. Alterations in dendritic spine structure or function can affect the processing of synaptic inputs. Seizures are implicated in excessive neuronal excitability, and these changes may constitute a compensatory response in the form of a homeostatic mechanism. However, the loss of excitatory input eventually affects inhibitory networks, which in turn may cause increased excitability and an inclination toward seizures.

An important issue are the mechanisms that are involved in dendritic spine pathology in epilepsy. The ECM constitutes a natural milieu for dendritic spines, and ECM remodeling may potentially influence epileptogenesis on many different levels, predominantly related to structural reorganization. Extracellular structural networks stabilize cellular and synaptic components. When pathology occurs, however, these stable components break down, which may lead to abnormal structural reorganization (i.e., dendritic spine loss or axonal sprouting) that promotes circuitry reorganization and epileptogenesis. Seizures lead to striking remodeling of the ECM, which may be essentially engaged in different aspects of epileptogenesis (Dityatev, 2010; Lukasiuk et al., 2011). Metalloproteinases, enzymes that modulate the ECM, grow to be important players in these processes. Evidence for a role of MMP-9 in physiological and aberrant synaptic plasticity and posttraumatic epileptogenesis is especially strong, in contrast to other MMPs (Lukasiuk et al., 2011).

Some evidence also comes from human studies. The critical role of MMP-9 in pathology following trauma is supported by clinical studies, in which elevated levels of MMP-9 were detected in cerebrospinal fluid from patients with severe traumatic brain injury (Grossetete et al., 2009). Prolonged seizures are also related to high serum MMP-9 levels in patients (Suenaga et al., 2008). MMP-9 is also upregulated in the cortex in patients with focal cortical dysplasia, a disorder associated with intractable cortical epilepsy (Konopka et al., 2013). Notably, the prominent upregulation of MMP-9 was observed mainly at postsynaptic sites (i.e., at dendritic spines).

The involvement of the MMP-9 proteolytic system has been widely studied in animal models of epilepsy. After traumatic brain injury, the activation of MMP-9 in lesioned cortex occurred within 1 day following trauma and remained elevated for 7 days after the initial insult (Hadass et al., 2013). Moreover, treatment with an MMP-9 inhibitor effectively attenuated MMP-9 activity, reduced brain lesion volume, and prevented neuronal loss and dendritic degeneration (Hadass et al., 2013). Similarly, after brain trauma, MMP-9 protein levels are increased compared with the contralateral cortex, with a peak 24 h following injury and elevations that persist for up to 1 week. Moreover, MMP-9 knockout mice have smaller traumatic brain lesion volumes (Wang et al., 2000).

**Figure 3:**
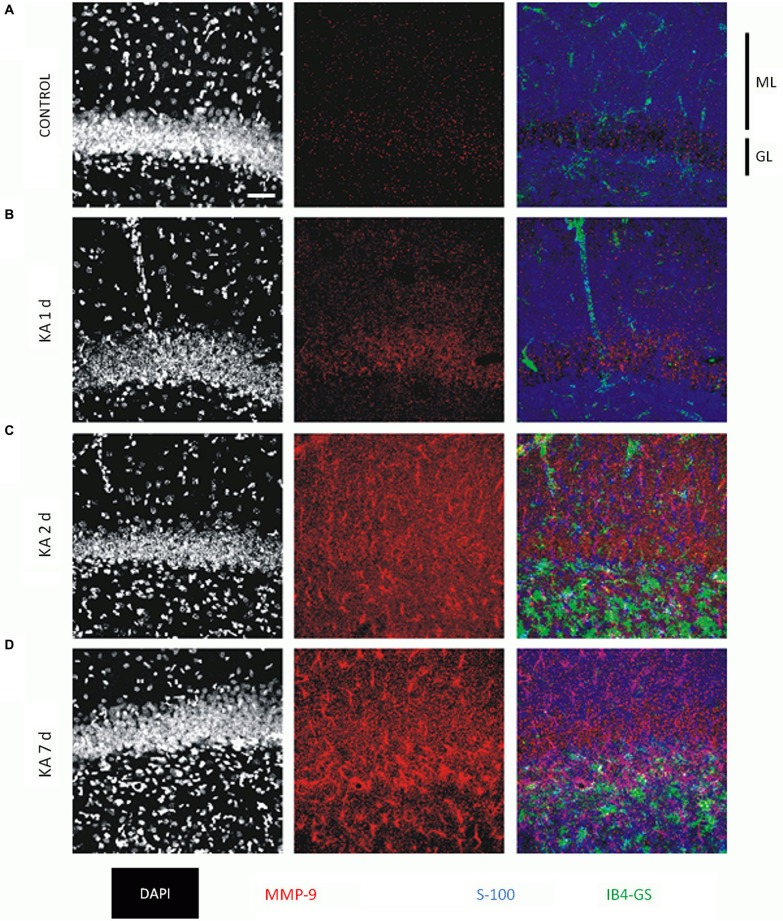
**MMP-9 immunoreactivity (IR) in the Dentate Gyrus (DG) of control animal and 1–7 days following intra-amygdalar kainic acid (KA) administration**. **(A)** Immediately after KA administration MMP-9 IR is low and distributed equally across DG layers. **(B–D)** MMP-9 IR increases after KA injection. **(C–D)** Note increased IR of MMP-9 predominantly in molecular layer (ML). **(D)** Seven days post KA administration neuronal MMP-9 reservoir is enriched with astrocytic pool. Confocal images from brain tissue labeled with nuclear marker (DAPI—white), MMP-9 antibody (red), astrocyte marker anitbody (S-100, blue), microglia marker antibody (IB4-GS, green). GL—granular layer, ML—molecular layer. Scale bar 50 μm. The experiment was performed by dr Grzegorz Wilczynski. For further reference, see Wilczynski et al. (2008).

MMP-9 is also induced during status epilepticus after treatment with kainate (Zhang et al., 2000; Szklarczyk et al., 2002; Jourquin et al., 2003; Konopacki et al., 2007) and pilocarpine (Kim et al., 2009). MMP-9 immunoreactivity (IR) is increased 24 h after kainic acid (KA) administration. IR is initially observed in neurons, and expression remains elevated for up to 7 days. Notably, MMP-9 IR is also observed in astrocytes (Figure 3). The increased expression and activity of MMP-9 has been shown to also occur with pentylenetetrazol (PTZ)-induced seizures (Michaluk et al., 2007; Rylski et al., 2009; Mizoguchi et al., 2011b). In these models, MMP-9 has been suggested to contribute to neuronal death (Jourquin et al., 2003; Kim et al., 2009), dendritic spine pruning (Szklarczyk et al., 2002), and the formation of aberrant synaptic contacts (Szklarczyk et al., 2002).

Strong evidence for a pivotal role of MMP-9 in epileptogenesis-related plasticity was provided by Mizoguchi and Wilczynski (Wilczynski et al., 2008; Mizoguchi et al., 2011a). Repeated treatment with PTZ produced kindled seizure, accompanied by enhanced MMP-9 activity and expression in the hippocampus (Mizoguchi et al., 2011a). The sensitivity to PTZ kindling was decreased in MMP-9 knockout mice (Wilczynski et al., 2008; Mizoguchi et al., 2011a). These mice also exhibited less severe seizures. In contrast, rats with constitutive neuronal MMP-9 overexpression that received PTZ treatment were more susceptible to seizures than wildtype animals. MMP-9 has been suggested to facilitate the development of seizures by affecting epilepsy-related synaptic plasticity. Seizure-induced MMP-9 expression was previously shown to be localized to dendrites and synapses and implicated in synaptic remodeling and mossy fiber sprouting, pathological structural phenomena associated with epilepsy (Szklarczyk et al., 2002; Michaluk and Kaczmarek, 2007; Gawlak et al., 2009). Indeed, MMP-9 protein levels and activity localized at synapses were strongly upregulated following intraperitoneal kainate treatment (Wilczynski et al., 2008). In the same study, unilateral kainate injections into the amygdala were used to induce status epilepticus. Twenty-four hours after seizure onset, spine density decreased in the DG on the injected side compared with the contralateral side in MMP-9 wildtype animals. In contrast, no difference in spine density was found between the injected and contralateral sides in MMP-9 knockout mice. Overall, status epilepticus-induced dendritic spine loss in the ML of the DG appears to be mediated by MMP-9 release from spines in response to seizures. In MMP-9 knockout animals the pruning of dendritic spines is abolished, despite the presence of stimulus. MMP-9 was also shown to be involved in mossy fiber sprouting and aberrant synaptogenesis in hippocampal epileptogenesis, in which neither of these phenomena develop in the absence of the enzyme. Interestingly, mossy fiber sprouting sites have been associated with the regrowth of dendritic spines in this region after status epilepticus (Isokawa, 2000).

MMP-9 activity was also considerably enhanced in other epilepsy models that do not involve neuronal cell loss (Baracskay et al., 2008; Gallyas et al., 2008; Sarkisova and van Luijtelaar, 2011). MMP-9 may be involved in generalized absence epilepsy, in which increased activity was found in WAG/Rij rats (Takacs et al., 2010). These rats spontaneously produce absence-like seizures caused by the hypersynchronic activity of thalamocortical and corticothalamic neurons and are commonly used as an animal model of human absence epilepsy (Coenen and Van Luijtelaar, 2003). Interestingly, the administration of doxycycline, an MMP inhibitor, aggravated epileptiform activity in WAG/Rij rats (Kovács et al., 2011). Matrix metalloproteinases were previously shown to act directly on NMDA and AMPA receptors (Michaluk et al., 2009; Gorkiewicz et al., 2010). This may, in turn, provide a sort of balanced homeostatic synaptic plasticity during seizures. NMDA and AMPA receptors are involved in the genesis of absence seizures (Coenen and Van Luijtelaar, 2003). Therefore, MMP-9 inhibition may increase the net level of excitability and increase absence-like epileptic activity in WAG/Rij rats (Takacs et al., 2010).

Considering the extant evidence, MMP-9 might play a dual role in epilepsy, with distinct roles in pathogenesis at various time-points after seizures (Michaluk and Kaczmarek, 2007). Aside from contributing to structural remodeling, MMP-9 may also be involved in maintaining homeostatic synaptic plasticity to counteract epileptic seizures. Fast activation of MMP-9 by seizures (i.e., within minutes) may induce spine loss (which is not observed in MMP-9 knockout mice) that initially alters neuronal excitability and reduces the effects of seizures. Sustained increases in MMP-9 levels (secondary to seizures, within days) may be hypothesized to push the balance toward the regrowth of dendritic spines and restore system balance but in effect contribute to long-term circuitry remodeling. Such aberrant plasticity may underlie epileptogenesis and lead to the formation of epileptic foci in the brain (Figure 2).

## Conclusions

Brain plasticity relies on modifications in synaptic connectivity that are driven by molecular changes in neurons and the ECM. The ability to change synaptic connections comprises alterations of dendritic spines at the morphological level. We reviewed the role of ECM metalloproteinase activity in physiological and pathological structural plasticity. We summarized the contribution of MMP-9 in the dynamic remodeling of the ECM via the cleavage of numerous extracellular substrates and its role in the formation, modification, and maintenance of dendritic spines. Importantly, MMP-9 may serve as an example of a proteolytic modifier of the ECM, thus supporting the concept of a tetrapartite synapse. Finally, we examined the morphological abnormalities of dendritic protrusions that are well known to be associated with neuropsychiatric disorders, particularly those that involve cognitive deficits. We reviewed evidence that implicates MMP-9 in aberrant synaptic plasticity and spine dysmorphology in neuropsychiatric disorders.

## Conflict of interest statement

The authors declare that the research was conducted in the absence of any commercial or financial relationships that could be construed as a potential conflict of interest.
